# Caveolins: targeting pro-survival signaling in the heart and brain

**DOI:** 10.3389/fphys.2012.00393

**Published:** 2012-10-05

**Authors:** Creed M. Stary, Yasuo M. Tsutsumi, Piyush M. Patel, Brian P. Head, Hemal H. Patel, David M. Roth

**Affiliations:** ^1^Department of Anesthesiology, Veterans Affairs San Diego Healthcare System, University of CaliforniaSan Diego, La Jolla, CA, USA; ^2^Department of Anesthesiology, The University of TokushimaTokushima, Japan

**Keywords:** Cav-1, Cav-3, membrane lipid raft, MLR, caveolae, neurogenesis, preconditioning

## Abstract

The present review discusses intracellular signaling moieties specific to membrane lipid rafts (MLRs) and the scaffolding proteins caveolin and introduces current data promoting their potential role in the treatment of pathologies of the heart and brain. MLRs are discreet microdomains of the plasma membrane enriched in gylcosphingolipids and cholesterol that concentrate and localize signaling molecules. Caveolin proteins are necessary for the formation of MLRs, and are responsible for coordinating signaling events by scaffolding and enriching numerous signaling moieties in close proximity. Specifically in the heart and brain, caveolins are necessary for the cytoprotective phenomenon termed ischemic and anesthetic preconditioning. Targeted overexpression of caveolin in the heart and brain leads to induction of multiple pro-survival and pro-growth signaling pathways; thus, caveolins represent a potential novel therapeutic target for cardiac and neurological pathologies.

## Introduction

Cardiac (i.e., ischemic heart disease, cardiomyopathy, congestive heart failure) and neuronal (i.e., stroke, traumatic brain injury, neurodegenerative disorders) diseases together represent a majority of mortalities (Hoyert, 2012) and sources of disability (Soni, 2011) in the United States and the developed and developing world. In survivors, these diseases are collectively associated with a high rate of rehabilitation and institutionalization, which translates to a significant cost and resource burden on the healthcare system (Soni, 2011). To date, clinical trials investigating pharmacologic therapies targeted at pro-survival pathways in the heart and brain have been largely ineffective in enhancing recovery and improving clinical outcome (Marler, 2007; Rose et al., 2010). This may be due to a limited ability to regulate pro-survival signaling that can occur in these organ systems following injury (Hicks et al., 1998; Biegon et al., 2004; Atkins et al., 2007, 2009; Rose et al., 2010). This review will highlight recent studies demonstrating that targeted augmentation of the subcellular microdomains that coordinate the trafficking and localization of signaling events restores multiple pro-survival and pro-growth signaling pathways specifically in the heart and brain, which may provide a new therapeutic avenue for treating a number of disease states affecting these organ systems.

## Membrane lipid rafts, caveolae, and caveolins

**Figure 1:**
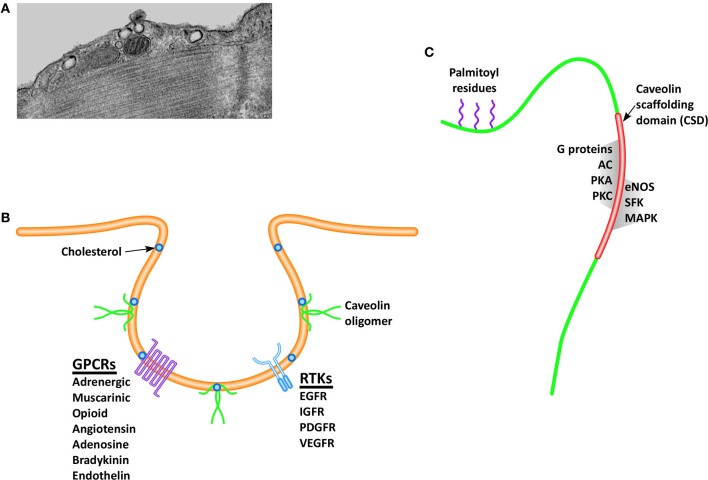
**Caveolae are invaginations of the cell membrane readily visualized via electron microscopy (A), which play a critical role in pro-survival and pro-growth signaling.** The formation and stabilization of caveolae depend on the presence of cholesterol and the structural protein caveolin, and serve to spatially localize signaling receptors **(B)**, including receptor tyrosine kinases (RTKs) and G-protein coupled receptors (GPCRs). Caveolin monomers **(C)**, contains a palmitic acid anchor that serves to stabilize the protein within the cell membrane, and a scaffolding domain, which is the binding site for many pro-survival and pro-growth molecules. (Definitions: EGFR, epidermal growth factor receptor; IGFR, insulin growth factor receptor; PDGFR, platelet derived growth factor receptor; VEGFR, vascular endothelial growth factor receptor; AC, adenylyl cyclase; PKA, protein kinase A; PKC, protein kinase C; eNOS, endothelial nitric oxide synthase; SFK, Src family kinases; MAPK, mitogen activated protein kinases).

Membrane lipid rafts (MLRs) are discreet microdomains of the cell membrane that concentrate and localize signaling molecules by providing a stable platform for protein anchoring. Stability of protein anchoring is afforded by the lipid composition of MLRs, which are rich in sphingomyelin, glycosphingolipids, and cholesterol. By promoting a favorable binding environment for cell signaling receptors and their downstream effectors, MLRs promote a variety of physiological functions such as cell surface signaling (Lisanti et al., 1994; Ostrom et al., 2001; Steinberg and Brunton, 2001; Ostrom, 2002; Williams and Lisanti, 2004), endocytosis (Anderson, 1993), calcium homeostasis (Fujimoto et al., 1992; Fujimoto, 1993; Scriven et al., 2002) and intracellular cholesterol transport (Murata et al., 1995; Smart et al., 1996). Caveolae (Figure 1A), morphologic invaginations of the cell surface, are subsets of MLRs containing sphingolipids and cholesterol (Figure 1B) that were first observed in 1953 by Palade (1953), but their role in cell-surface signaling has only been realized in the past two decades. By regulating the intracellular trafficking and delivery of cholesterol to the cell membrane, caveolar formation and stabilization is dependent on the key structural protein, caveolin (Smart et al., 1999).

Caveolins (Figure 1C) are a family of proteins approximately 17–24 kDa in size that exist in three isoforms (Cav-1, -2, and -3), all containing an invariant hydrophobic central domain that promotes insertion in lipid bilayers in a hairpin loop manner, with both the COOH and NH_2_ termini facing the cytoplasm (Dupree et al., 1993; Dietzen et al., 1995; Monier et al., 1995; Tang et al., 1996). Initial gene knockout (KO) studies provided evidence that Cav-1 is essential for formation of caveolae in endothelial cells, fibroblasts, and pneumocytes (Razani et al., 2001), whereas Cav-2 plays an unclear but likely supportive role by forming hetero-oligomers with Cav-1 (Monier et al., 1995; Razani et al., 2001). Cav-3 KO mice are viable but subject to skeletal and cardiac myopathies (Hagiwara et al., 2000; Galbiati et al., 2001), belying a critical role for Cav-3 in muscle (Tang et al., 1996; Galbiati et al., 1998). However, all three caveolin isoforms have been subsequently demonstrated in the central nervous system (CNS) (Zschocke et al., 2002; Shin et al., 2005), suggesting a greater degree of tissue ubiquity for Cav-3, despite early indications of specificity to muscle. Similarly, Cav-1 expression has subsequently been demonstrated in both atrial (Volonte et al., 2008) and ventricular myocytes (Cho et al., 2010). Caveolins are also present in non-caveolar rafts, which exist as planar, transient membrane microdomains, where they are complexed with glycophosphatidylinositol-anchored signaling proteins (Boscher and Nabi, 2012), implying a spatial role in cell-surface and intracellular signaling cascades. In fact all three caveolin isoforms contain a conserved “caveolin scaffolding domain” (CSD, Figure 1C), which functions as the physical binding site for a number of signaling molecules.

## Caveolins coordinate multiple signaling pathways in the heart

A number of signaling molecules localize to the MLR by aggregating with the CSD on the C-terminal end of the caveolin protein (Figure 1C). G-protein coupled receptors (GPCRs) comprise a large superfamily of transmembrane signaling receptors that transduce extracellular molecules (e.g., endothelin, bradykinin, serotonin, angiotensin-1, opioid, adenosine, and adrenergic) to intracellular signals. It has been proposed that compartmentalization of these receptors and other proteins in plasma membrane microdomains may promote and regulate intracellular signaling (Ostrom, 2002; Ostrom et al., 2002; Insel et al., 2005). Evidence to support the interaction between GPCRs within MLRs came from observations that the β2 adrenergic receptor localized to lipid-rich buoyant fractions and within caveolae (Ostrom et al., 2000; Rybin et al., 2000). Compartmentalization of signaling was further suggested by experiments demonstrating that the inactive Gα subunit may concentrate in caveolae and associate with the CSD (Oh and Schnitzer, 2001), while activation of the subunit causes Gα to dissociate and remain separate from caveolae (Li et al., 1995). Moreover, endocytosis of ligand-bound GPCRs occurs via a caveolae-dependent mechanism, which in turn initiates or terminates signaling (Escriche et al., 2003; Rapacciuolo et al., 2003).

Endothelial nitric oxide synthase (eNOS) is involved in the control of many important functions, such as angiogenesis, vasorelaxation, and permeability. Mice deficient in Cav-1 display increased nitric oxide (NO) production from increased eNOS activity (Razani et al., 2001) with resultant increased vascular permeability in mouse lung endothelium (El-Yazbi et al., 2006), both of which are reversed by injection of a truncated Cav-1 peptide containing the CSD. Inhibition of eNOS by caveolin occurs within the systemic vascular endothelium, whereby eNOS activity does not respond to regulatory signals, NO levels remain increased, and basal cGMP levels remain elevated in rings of aorta from Cav-1 deficient mice (Drab et al., 2001; Razani et al., 2001). A role for Cav-1 as a direct negative regulator of eNOS has been demonstrated whereby application of peptide containing the caveolin CSD *in vivo* resulted in selective inhibition of NO (Bucci et al., 2000). However, an obligatory role for caveolin on eNOS function has also been demonstrated, whereby depletion of cholesterol resulted in disruption of agonist-stimulated NO release in endothelial cells (Blair et al., 1999). While a role for Cav-1 in endothelial NO production is evident, Cav-3 likely also plays a critical role in NO signaling in skeletal and cardiac tissue. Feron et al. (1996) first demonstrated a tissue-specific differential pattern of caveolin/eNOS co-localization, whereby eNOS was observed to complex with Cav-1 in endothelial cells, but with Cav-3 in cardiac myocytes, while Segal et al. (1999) demonstrated co-localization of Cav-3 and neuronal NOS (nNOS) in skeletal muscle. Application of oligonucleotide coding for the Cav-3 CSD to permeabilized cardiomyocytes specifically inhibited a cholinergic-mediated decrease in myocyte chronotropy and blunted elevations in cGMP, demonstrating modulation of cardiac myocyte function via the interaction of Cav-1 and eNOS (Feron et al., 1998). Global overexpression of Cav-3 was subsequently shown to result in severe cardiomyopathy and muscular dystrophy accompanied by downregulation of NOS (Aravamudan et al., 2003). Modulation of cardiac and skeletal muscle angiogenesis and vasoreactivity by the interaction of Cav-3 and NOS may in part explain the phenotype of Cav-3 deficient mice, which also includes both skeletal and cardiac myopathies (Galbiati et al., 2001).

Another fundamental regulator of cell growth and differentiation is protein phosphorylation via intracellular kinases, downstream effectors of cell surface receptor binding. Phosphatidylinositol 3-kinase (PI3K) can be activated by GPCRs or tyrosine kinase receptors, and is intimately involved in cell growth and survival through activation of the anti-apoptotic Akt pathway. In cultured skeletal myocytes, disruption of MLR's impairs cell survival via inhibition of PI3K/Akt (Smythe and Rando, 2006). Furthermore, PI3K regulates insulin signaling, whereby caveolin depletion alters insulin resistance in skeletal muscle and adipose tissue (Cohen et al., 2003). Mitogen-activated protein kinases (MAPK) represent another class of protein kinases that regulate cell proliferation (Rose et al., 2010). Increased MAPK activity downregulates Cav-1 mRNA and protein levels, and overexpression of Cav-1 inhibits the MAPK signaling pathways, an inhibition that is dependent on the CSD (Engelman et al., 1998). Finally, tyrosine kinases are thought to localize to MLRs and to interact with Cav-1 (Li et al., 1996). Phosphorylation of Cav-1 occurs via the non-receptor tyrosine kinase Src (Volonte et al., 2001), which can induce muscle degeneration and inflammatory gene expression if Cav-1 expression and localization is disrupted (Smythe and Rando, 2006), but which has been implicated in myocardial protection from ischemia/reperfusion (IR) injury (Patel et al., 2007). Moreover, vascular endothelial growth factor receptor has been reported to interact with Cav-1, and initiation of angiogenesis via tyrosine kinase activation is dependent on the presence of Cav-1, underlying a significant role for caveolin in the regulation of cell growth and survival (Feng et al., 1999; Labrecque et al., 2003).

## Caveolin-3 regulates cardiac hypertrophy

Stress on the heart produces pathogenic cell growth, whereby hemodynamic overload induces an initial hypertrophic response modulated by several signaling pathways that affect gene expression, apoptosis, inflammation, and growth factor signaling but which ultimately ends in ventricular dilation and failure (Rohini et al., 2010). Genetic deletion of Cav-1 results in a progressive biventricular cardiomyopathy, with sustained activation of MAPK, Akt and eNOS, and diminished ATP content in the heart (Cohen et al., 2003). A recent study by Cruz et al. (2012) suggested that elevated pulmonary pressures in Cav-1 deficient mice contributed to eNOS uncoupling, whereby chronic hypoxia lead to right ventricular hypertrophy, while endothelial-specific upregulation of Cav-1 ablated these changes. Given that the expression of Cav-1 is required for caveolar formation in non-muscle cells, and caveolin-3 drives caveolae formation in cardiac and skeletal muscle, a Cav-1/3 double KO mouse was generated by Park et al. (2002) in order to investigate phenotypic cardiac changes in mice completely lacking morphologic caveolae. Cav-1/3 double KO mice displayed a severe cardiomyopathy, with a dramatic increase in left ventricular (LV) wall thickness, decreased LV fractional shortening, disorganized, and degenerated cardiomyocyte ultrastructure, chronic interstitial fibrosis and inflammation, and augmentation of ANP expression as compared with Cav-1-KO, Cav-3 KO, and wild-type mice (Park et al., 2002). While genetic deletion of caveolin results in hypertrophy, global overexpression of Cav-3 also results in a cardiomyopathic phenotype characterized by degeneration and fibrosis (Aravamudan et al., 2003), and as well as dystrophic skeletal muscle phenotype (Galbiati et al., 2000). In contrast, Koga et al. (2003) demonstrated that adenovirus-mediated overexpression of Cav-3 in isolated cardiomyocytes provided protection from phenylephrine-induced hypertrophy, suggesting that modulation of Cav-3 specifically in cardiac myocytes may provide cardioprotection against dynamic overload. Additionally, natriuretic peptides that can modulate cardiac hypertrophy by regulating the adaptive response of the heart to hemodynamic overload via diuretic, natriuretic and vasodilatory action, are associated with caveolae and Cav-3 (Newman et al., 1991; Doyle et al., 1997). Subsequently, we demonstrated that adenovirus-mediated overexpression of Cav-3 in cardiomyocytes augments natriuretic peptide expression and Akt phosphorylation, which was blocked by PI3K inhibition and caveolar disruption (Horikawa et al., 2011). Given the potential for Cav-3 to modulate cardiac hypertrophy, we next tested whether targeting Cav-3 specifically to cardiac myocytes *in vivo* would alter natriuretic peptide and Akt signaling and attenuate the development of cardiac hypertrophy induced by hemodynamic overload. We developed a cardiac-targeted (α-myosin heavy chain promoter) Cav-3 overexpression system that caused a greater than twofold increase in Cav-3 specifically in cardiac myocytes and subjected the mice to transverse aortic constriction (TAC) for 1 month, to induce cardiac hypertrophy. Mice with cardiac-specific overexpression of Cav-3 exhibited augmentation of natriuretic peptide expression and nuclear Akt phosphorylation, resulting in reduced cardiac hypertrophy following TAC, with improved cardiac function and increased survival suggesting a potential therapeutic role for Cav-3 in heart failure (Horikawa et al., 2011).

## Caveolin-3 regulates cardiac ion channels in the heart

In addition to potential therapeutic modalities targeting the mitogenic functions of caveolin, recent evidence suggests that Cav-3 expression is a critical element in modulation of membrane potential via direct interaction with ion channels (O'Connell et al., 2004; Balijepalli et al., 2006; Maguy et al., 2006; Balijepalli and Kamp, 2008; Balse et al., 2012). In the cardiac myocyte, it is established that one mechanism for Ca^2+^ influx occurs via opening of voltage-gated Na^+^ channels which initiates depolarization through the cardiac sarcolemma, inducing activation of voltage-gated L-type Ca^2+^ channels (LCC), resulting in Ca^2+^ influx into the cytosol and subsequent contraction. However, current evidence suggest a more complicated system of basal oscillating “sparks” and “puffs” of cytosolic Ca^2+^ mediated by ligand-gated receptors (Cheng and Lederer, 2008) and contraction-independent LCCs (Makarewich et al., 2012). Dysregulated Ca^2+^-flux through LCC is thought to play a role in the development of cardiac hypertrophy, and early evidence suggests flux through LCC in Cav-3 rich microdoamins as a locus for this pathologic growth via the calcineurin-nuclear factor of activated T-cell signaling cascade (Makarewich et al., 2012). Cav-3 also is involved in the protein kinase A-dependent stimulation of T-type Ca^2+^ channels, which are re-expressed in the adult heart during hypertrophy and associated with cardiac dysfunction in heart failure (Markandeya et al., 2011). A recent study by Guo et al. (2011) suggests that Cav-3 may modulate cardiac hypertrophy and contractility via the GPCR subunit Gαq, which is responsible for activation of phospholipase C-mediated Ca^2+^ flux in the cardiomyocyte. Moreover, Cav-3 appears necessary to modulate adrenergic activation of protein kinase A and phosphorylation of phospholambam-independently of LCC in ventricular myocytes (Macdougall et al., 2012). Caveolae appear to contribute to regulation of excitation-contraction cycling through modulation of both Ca^2+^ (Lohn et al., 2000; Bergdahl et al., 2003; Kwiatek et al., 2006; Besse et al., 2011) and K^+^ flux (Martens et al., 2001; Wang et al., 2005). Voltage-gated K^+^ channel localizes to caveolae (Martens et al., 2001), while depletion of cholesterol impairs maintenance of intracellular (K^+^), leading to destabilization of membrane polarization (Fagan et al., 2000). Conduction of depolarization throughout the heart also appears dependent on caveolins, as Cav-3 KO animals display disorganization of the T-tubule complex (Galbiati et al., 2001), and mutations in the Cav-3 gene have been identified in patients with congenital long-QT syndrome (Vatta et al., 2006). Modulation of caveolin levels specifically in the heart therefore represents a potential mechanism to restore the loss in contractility and/or rhythmicity that occurs in pathogenic remodeling states such as congestive heart failure.

## Caveolin-3 protects the heart from ischemia/reperfusion injury

Caveolins have been shown to play a fundamental role in the phenomenon of myocardial preconditioning, whereby an initial priming stimulus confers cytoprotection to a subsequent bout of IR. Historically, the first preconditioning stimulus described in 1986 by Murry et al. (1986) was a short period of sub-lethal ischemia which they termed ischemic preconditioning (IPC). Subsequently both opioids (Schultz et al., 1997) and volatile anesthetic agents (Cason et al., 1997; Kersten et al., 1997), as well as other pharmacologic agents that activate GPCRs, were shown to confer a similar protective advantage. Preconditioning occurs in a biphasic pattern: an early phase, which occurs within minutes of the conditioning stimulus via post-translational modification of pre-existing proteins, and lasts 1–2 h, and a delayed phase occurring 12–24 h afterwards mediated by *de novo* synthesis of pro-survival signaling components (Tonkovic-Capin et al., 2002). Many of the signaling mediators involved in cardiac protection including the Gα subunit of heterotrimeric G-proteins, Src kinases, PI3K, eNOS, MAPK, and the end-effector of IPC/APC, the K_*ATP*_ channel, are known to localize to MLRs (Krajewska and Maslowska, 2004). An early observation made by our laboratory was that IPC and APC induce alterations in the sarcolemmal membrane ultrastructure of the myocyte, increasing the number of caveolae (Patel et al., 2006, 2007), and that the intrinsic ability of rat cardiomyocytes to undergo opiod-induced preconditioning depended on the presence of caveolae and localization of the δ-opiod receptor with Cav-3 (Patel et al., 2006). In followup studies, we utilized both *in vitro* and *in vivo* models to demonstrate that early-phase APC operated via a Src-dependent manner, whereby depletion of caveolae, deficiency of Cav-1 or inhibition of Src resulted in abolishment of the cytoprotective effects of the early-phase of isoflurane-induced preconditioning (Patel et al., 2007). Additionally, we observed a decreased number of myocardial caveolae in Cav-3 deficient mice, and that such mice cannot be “preconditioned” by IPC or APC (Horikawa et al., 2008; Tsutsumi et al., 2008). Given these findings, we tested the hypothesis that cardiac-specific overexpression of Cav-3 would increase caveolae, and induce an innate level of cardiac protection similar to IPC. Adenovirus-mediated Cav-3 overexpression in cardiomyocytes increased caveolar formation and induction of IPC-mediated pro-survival kinases (Tsutsumi et al., 2008). *In vivo* studies supported these findings: transgenic mice with cardiac-specific (α-myosin heavy chain promoter) Cav-3 overexpression demonstrated increased myocardial caveolar formation, augmentation of pro-survival kinases, and exhibited innate cardioprotection with improved cardiac function at a level similar to that achieved through IPC (Tsutsumi et al., 2008).

While early-phase preconditioning appears to involve the phosphorylation of preexisting proteins, delayed APC induces gene and protein expression changes that ultimately lead to the induction of various mediators, including induction of NOS (Chiari et al., 2005) and synthesis of glucose-transporter 4 (Glut-4, Nishino et al., 2004). While a clear role for Cav-1 and Cav-3 exists in the induction of the early-phase of preconditioing, we sought to investigate whether either caveolin isoform contributed to the regulation of protein expression during the delayed-phase of preconditioning. Using an *in vivo* approach, we subjected both Cav-1 and Cav-3 deficient mice to isoflurane exposure, followed by 24 h of recovery prior to IR, and observed that both the formation of morphologic caveolae and induced cardioprotection during delayed IPC was a Cav-3 dependent event (Tsutsumi et al., 2010). The time-course of cardioprotection appeared to coincide with translocation of Glut-4 to caveolae, with co-localization of Cav-3/Glut-4 complexes within the LV. Moreover, while early disruption of caveolae in cardiomyocytes did not disrupt delayed cardioprotection, disruption of caveolae following 24 h after isoflurane exposure (but just prior to IR) did, implicating morphologic caveolae as necessary mediators in the late-phase of APC (Tsutsumi et al., 2010). Collectively these studies suggest that Cav-3 is both necessary and sufficient to promote the formation of caveolae and to induce both early and delayed cardiac protection from IR injury. Interestingly, a recent study by Waldenström et al. demonstrated that cardiomyocytes secrete MLR-like microvescicle “exosomes” that contain Cav-3 (Waldenstrom et al., 2012), raising the intriguing possibility that control of cell signaling may not be spatially restricted to a single cell, and that caveolins may play a role in inter-cellular and possibly inter-organ communication. Although our understanding of the mechanisms by which caveolins and MLRs provide cardiac protection remains incomplete, it appears that caveolin-induced enhancement of pro-survival and pro-growth pathways offers a promising avenue for clinical trials in the treatment of multiple diseases of the heart.

## Caveolin-1 regulates neuronal signaling

**Figure 2:**
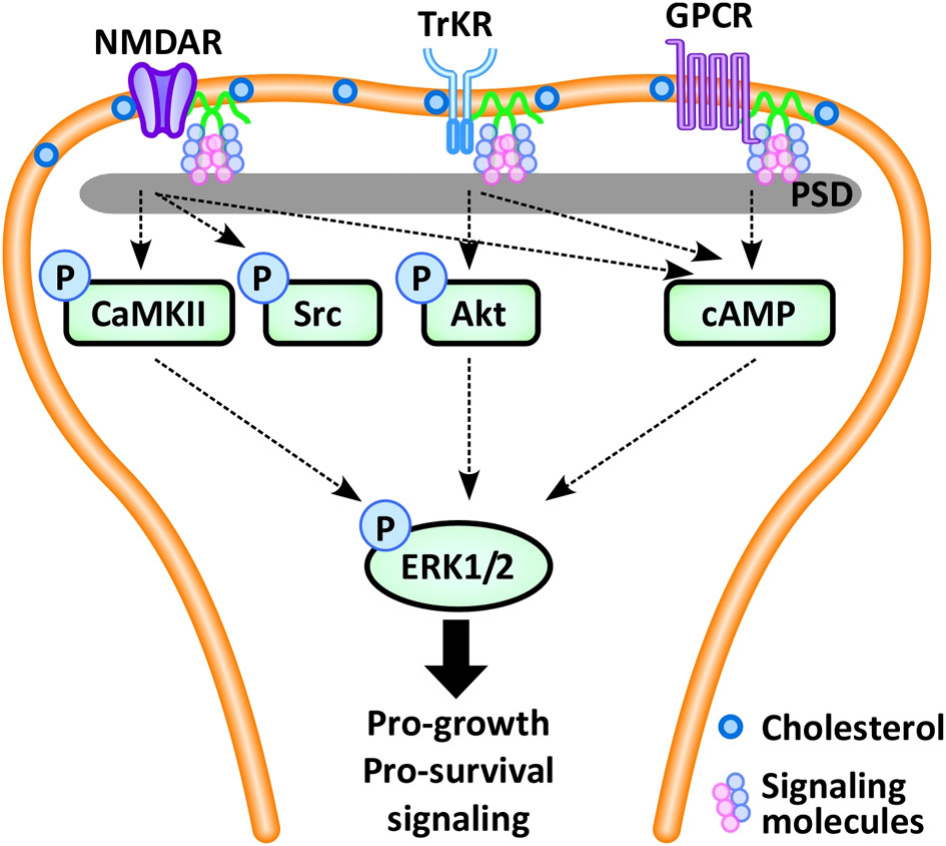
**In the neuron, pro-survival signaling occurs via multiple synaptic signaling receptors, including the NMDA glutamate receptor (NMDAR), the neurotrophin-activated tyrosine kinase B receptor (TrkR), and G-protein coupled receptors (GPCRs), which converge via intracellular protein kinases (CaMKII, Src, Akt, cAMP) to phosphorylate extracellular-signal regulated kinase 1/2 (ERK1/2), leading to expression of pro-survival and pro-growth genes.** These pro-survival receptors localize and concentrate within the post-synaptic density (PSD), a region enriched in caveolins, cholesterol and glycosphingolipids. (Definitions: CaMKII, calcium/calmodulin-dependent protein kinase II; cAMP, cyclic adenosine monophosphate).

Although it is now recognized that all three caveolin isoforms exist in neurons, a discreet absence of caveolae likely contributed to the late recognition that caveolins are not only present within neuronal cell membranes but also contribute to neuronal pro-survival signaling (for review see Stern and Mermelstein (2010). Caveolin-dependent signaling appears to be mediated by spatial localization of relevant molecules, either through clustering and sequestering, thereby exerting both positive and negative regulation of signaling (Galbiati et al., 1998). For example, while Cav-1 knockdown impedes mobilization of intracellular Ca^2+^ by serotonin 2A subtype-receptor (Bhatnagar et al., 2004), Cav-1 appears to internalize and attenuate the activity of the Gαs-coupled dopamine receptor (Kong et al., 2007). Localization of signaling is important in the neuron, as extra-synaptic activation may induce excitotoxicity (Figure 2), promoting cell death (Hardingham and Bading, 2003). In neurons, Cav-1 co-localizes with the synaptic-specific protein PSD-95 (Boeckers, 2006), and with the pro-survival signaling receptors such as the NMDA glutamate receptor subtype 2A, indicating spatial regulation of neuronal signaling (Head et al., 2008). Targeting of caveolin to the synapse suggests the pivotal role they are thought to play in synaptic development of retinal ganglion cells (Mauch et al., 2001) and at the neuromuscular junction (Willmann et al., 2006). Cav-1 is expressed in multiple types of neurons, including hippocampal and dorsal root ganglion neurons (Galbiati et al., 1998; Peiro et al., 2000; Bu et al., 2003; Gaudreault et al., 2004), and Cav-1 deficient mice exhibit neurological abnormalities including, abnormal spinning, muscle weakness, reduced activity, and gait abnormalities (Trushina et al., 2006). Additional work demonstrates that Cav-1 KO mice exhibit an early aging phenotype, which includes loss of synapses, enhanced astrogliosis and changes in the cerebrovasculature (Head et al., 2010). Interestingly, Cohen et al. note in unpublished observations that Cav-1 KO mice display an increased sensitivity to inhaled anesthetics (Cohen et al., 2003), resulting in profound bradycardia given a standard level of anesthetic. Whether these observations are mediated by a central or peripheral mechanism is unclear. One intriguing phenomenon proposed by Lucchinetti et al. (2008) considers the preferential partitioning of halogenated ethers to amphiphilic interfaces [such as lipid rafts (Morgan et al., 2004)] as a significant event in cell signaling, whereby exposure to anesthetics may alter the interaction of specific protein-protein and/or protein-lipid interactions, thereby resulting in remodeling of the signaling architecture within the cell. As noted above, we have observed this in cardiomyocytes (Patel et al., 2007), whereby exposure to isoflurane resulted in an increase in morphological caveolae, supporting a mechanism where halogenated ethers alter the physiochemistry of the cell membrane. However, given that neurons do not exhibit morphologic caveolae it remains unclear whether MLRs in fact play a significant role in the anesthetic effects of halogenated ethers in the CNS. Currently this area has not been investigated and remains largely unknown.

Similar to myocardium, caveolins in neurons coordinate GPCRs, ion channels, and downstream kinase activation. Pro-survival signaling in neurons occurs following activation of a number of synaptic receptors, including GPCRs, the neurotrophin-activated tyrosine kinase B (TrkB) receptor, and the glutamate receptor, which share the common final pathway of phosphorylation of the cAMP response element binding protein (CREB), initiating transcription of pro-growth genes (Figure 2). Cav-1 and MLRs can regulate estrogen GPCR signaling (Mermelstein, 2009), whereas Cav-3 inhibition leads to loss of the estrogen-mediated inhibition of L-type Ca^2+^ channel-dependent CREB phosphorylation (Boulware et al., 2007). This latter effect may lead to Ca^2+^ flux and potentially neuromuscular contraction, synaptic transmission or upregulation of gene expression. Ionotropic receptor, TrkB-receptor, and GPCR-mediated neurotransmission appear to be caveolin-dependent (Head et al., 2008, 2011; Francesconi et al., 2009; Takayasu et al., 2010). Positive regulation of 5-HT_2*A*_R, a subtype of GPCR, likely occurs through facilitating binding between the receptor and Gαq subunit (Bhatnagar et al., 2004). Similarly, the activation of the NMDA receptor appears to be mediated by both Cav-1 and Cav-3 via controlled coordination of the Gαq subunit, with subsequent modulation of the pro-growth pathway ERK1/2 (Head et al., 2008). For example, we demonstrated that loss of Cav-1 expression disrupts NMDA receptor signaling and attenuates pro-survival Src and ERK1/2 phosphorylation in response to NMDA or simulated ischemia (Head et al., 2008, 2011).

## Caveolin-1 protects neurons from ischemic injury and promotes dendritic growth

Cerebral ischemia decreases the integrity of cell membranes and thus may disrupt caveolin-associated signaling complexes. A neuroprotective role for Cav-1 was first demonstrated in Cav-1 KO mice, which had a larger cerebral infarct size in response to ischemia (Jasmin et al., 2007) that may have been due to alterations in the permeability of the blood brain barrier (Gu et al., 2012) secondary to Cav-1 related translocation of the tight-junction protein claudin-5 (Liu et al., 2012). It was subsequently demonstrated that intracerebral hemorrhage resulted in elevated inflammatory mediators and a larger region of neuronal cell death in Cav-1 KO mice (Chang et al., 2011). This may be due to the complex role that signaling receptors are thought to play in the developing brain (Li et al., 1998), whereby disruption of MLRs results in multiple signaling deficiencies (Mauch et al., 2001; Willmann et al., 2006). Our laboratories corroborated the essential role of Cav-1 in phosphorylation of Src and ERK1/2 and IPC, by demonstrating that Cav-1 deficient neurons were unable to undergo preconditioning, while return of Cav-1 restored the capacity of the neuron to undergo the cytoprotective effect (Head et al., 2008, 2011). In addition to greater sensitivity to ischemia, mice deficient in Cav-1 have a CNS pathology similar to that exhibited in neurodegenerative diseases, including altered glutamate receptor signaling (Head et al., 2008; Francesconi et al., 2009; Takayasu et al., 2010), and impaired cholinergic function (Trushina et al., 2006; Jasmin et al., 2007; Gioiosa et al., 2008).

A role for caveolin-associated neurodegenerative disorders may be found in the lipid composition of the MLR (Parton, 1994), in particular GM-gangliosides, which are concentrated within MLRs and have been associated with autoimmune disorders such as lupus and Guillain-Barré syndrome (Bansal et al., 1994), in addition to being implicated in the development of dementia (Bansal et al., 1994). Interestingly, in post-mortem samples of human hippocampus and cortex in patients with Alzheimer's disease, cytospatial distribution of Cav-1 and the voltage-dependent anion channel (VDAC) is increased (Ramirez et al., 2009). Since the VDAC is thought to play a role in Alzheimer's-associated amyloid-β neurotoxicity (Ferrer, 2009), caveolins may play a prominent role in the modulation of amyloid precursor protein processing, and thus the development and progression of Alzheimer's disease. For example, functional Cav-1 plays a role in HIV-related accumulation of amyloid-β (Andras et al., 2012), while Cav-1 deficient mice develop a pathological phenotype similar to that of Alzheimer's disease (Head et al., 2010). Aging increases the likelihood of dementia and Alzheimer's disease, and localization of synaptic signaling components in neuronal MLRs is reduced in brains from aged WT and young Cav-1 KO mice (Head et al., 2010). Clearly, more research is needed on the regulatory role of caveolins/MLR on the activity of key secretases involved in amyloid processing.

Functional recovery from brain injury (i.e., stroke or trauma) and neurodegeneration is limited by a reduction in pro-survival and pro-growth signaling (Atkins et al., 2007), which increases neuronal loss, impairs brain repair, and increases functional deficits. This may explain why exogenous pharmacologic interventions targeting pro-survival pathways individually have been relatively ineffective. We have demonstrated that loss of Cav-1 decreases expression of MLRs, neurotransmitter and neurotrophin receptors, impairs neurotransmitter transduction, and attenuates pro-survival signaling (Head et al., 2008, 2011). A recent study (Lim et al., 2011), demonstrating that administration of exogenous gangliosides, a component of the MLR, resulted in augmentation of neurotrophic signaling in rat hippocampal and human neuronal cell lines, providing evidence that restoration of signaling may promote neuronal growth and improve functional outcome. However non-specific augmentation in pro-growth signaling may promote neoplastic processes, as there is evidence that the development of glioma is associated with increased Cav-1 production in astrocytes (Parat and Riggins, 2012). To this end, our laboratory utilized the neuron-specific promoter synapsin to overexpress Cav-1, which led to enhanced expression of MLRs, neurotransmitter and neurotrophin receptors specifically in neurons (Head et al., 2011). This resulted in an increase in pro-survival and pro-growth signaling molecules, translating to dendritic growth and arborization, even in the presence of inhibitory mediators. These promising results demonstrate that restoration of pro-survival and pro-growth signaling may be both necessary and sufficient to provide a therapeutic benefit in the functional recovery from brain injury and neurodegeneration.

## Conclusions

In the years following George Palade's first observations of caveolae nearly 60 years ago, diffusion-based fluid-mosaic models of the cell surface membrane (Singer and Nicolson, 1972) could not account for the speed and fidelity with which intracellular signaling occurs. It is clear now that the complexity of orchestrating multiple sub-cellular signaling events is accomplished by the focal concentration of signaling moieties within the MLR. This highly organized structure is dependent on caveolins, which facilitate and coordinate the spatial and temporal organization necessary to achieve multiple, simultaneous signaling activities. However, the model that caveolin is the only structural protein necessary for the formation of caveolae is becoming more complex with the description of the “cavin” family of proteins and other membrane proteins such as pacsin-2/syndapin-2, which are now known to play a key role in the formation and function of caveolae (Hansen and Nichols, 2010; Briand et al., 2011). Future work will need to focus on how the interaction of caveolins, cavins and other membrane structural proteins in and out of caveolar microdomains influences the potential pro-survival role of caveolins in the heart and brain.

By augmenting the expression of caveolin in the heart and brain, multiple pro-survival and pro-growth pathways are simultaneously upregulated, which is sufficient, and likely necessary, for inducing cardiac and neuroprotection. It remains to be resolved how control of caveolin expression can be harnessed in a clinical fashion to produce a beneficial therapeutic outcome. In addition to developing a gene delivery vehicle that is safe and efficacious in patients, post-transcriptional regulators such as RNAses and micro-RNAs may be able to be engineered to affect the gene expression machinery to augment potential protein therapies. Micro-RNAs (“miRs”) are small non-coding RNAs that serve to regulate post-transcriptional gene expression by binding to mRNA and repressing translation or degrading mRNA entirely. They are highly abundant in the brain and have been known to localize to subcellular regions such as dendritic spines and can affect spine structure/morphology, function, and synaptic plasticity (Gao et al., 2010; Saugstad, 2010). Although cell-specific genetic manipulation *in vivo* via viral vector delivery (e.g., adenoviral, adeno-associated viruses) has great therapeutic/translational potential, the use of small molecules to induce cell specific enhancement in mRNA translation will allow us to achieve alterations in protein expression-independent of viral vectors. An example is the use of complementary inhibitors of miRs, termed “antagomirs.” In regard to caveolin-specific miR inhibition, one group showed that the use of antagomirs to block Cav-1 associated miRs (miRs 103/107) and subsequently enhance Cav-1 protein expression reversed a type II diabetic phenotype (Trajkovski et al., 2011). Because caveolins afford robust protection against ischemic injury in both cardiac and neuronal tissue, identifying caveolin miRs specifically in the heart, brain, and spinal cord may allow us to design effective antagomirs with significant pharmaceutical implications. Given the potential of caveolin therapy to augment pro-growth signaling pathways, technologies to control the temporal expression of caveolins may be necessary as an “on–off” switch to prevent neoplasia. Although many aspects of harnessing the spatial and temporal regulation of cell signaling by caveolins remain to be determined, the studies described above represent a promising new therapeutic approach in which targeting cell survival signaling via caveolin expression may prove to not only augment existing pharmacologic modalities aimed at individual pro-survival pathways, but may alone be sufficient to promote cell survival and growth in a number of diseases of the heart and brain.

### Conflict of interest statement

The authors declare that the research was conducted in the absence of any commercial or financial relationships that could be construed as a potential conflict of interest.
